# Ultrasound performance in pediatric deep soft-tissue tumor characterization

**DOI:** 10.1038/s41598-023-48931-7

**Published:** 2023-12-13

**Authors:** Cong Li, Wenyi Chen, Ya Jin, Hong Xu, Hong Luo

**Affiliations:** 1 Department of Ultrasonography, West China Second University Hospital, Sichuan University, Chengdu, 610000 Sichuan China; 2 Department Key Laboratory of Birth Defects and Related Diseases of Women and Children, West China Second Hospital, Sichuan University, Chengdu, China

**Keywords:** Paediatric research, Cancer

## Abstract

This study investigated the performance of ultrasonography in diagnosing deep soft-tissue tumors and tumor-like lesions in children with histological results. Demographic information and ultrasound characteristics of benign and malignant masses were statistically analyzed. Three radiologists (Radiologists 1, 2, and 3) independently reviewed the ultrasonography studies while being blinded to the medical history and other imaging findings. The 82 lesions included in the study were histopathologically classified as malignant (n = 25) or benign (n = 57). No statistically significant differences were observed between the benign and malignant subgroups regarding age (p = 0.059), sex (p = 1.0), disease course (p = 0.812), presence or absence of symptoms (p = 0.534), maximum diameter (p = 0.359), margin (p = 1.0), calcification (p = 0.057), or blood Adler type (p = 0.563). However, statistically significant differences were observed between the benign and malignant subgroups in terms of isolated or Multiple occurrences (p < 0.001), history of malignancy (p < 0.001), shape (p < 0.001), and echogenicity (p < 0.001). Parameters such as tumor shape (p = 0.042, OR = 6.222), single or multiple occurrences (p = 0.008, OR = 17.000), and history of malignancy (p = 0.038, OR = 13.962) were identified as independent predictors of benign and malignant tumors. The diagnostic sensitivities evaluated by the three radiologists were 68.0%, 72.0%, 96.0%, respectively, while the specificities were 77.2%, 82.5%, 77.2%, respectively. Ultrasound demonstrates good performance in the diagnosis of benign deep lesions such as hemangiomas/venous malformation and adipocytic tumors. Multiple irregular morphologies and a history of malignancy were identified as independent risk factors for malignant masses. The experience of radiologists in recognizing specific tumors is important. Careful attention should be paid to masses with ambiguous ultrasound features, as well as small lesions.

## Introduction

Soft-tissue masses are common in children, although deep lesions are considered rare. However, malignant soft-tissue tumors disproportionately affect children, accounting for 7.4% of all tumors, compared to only 1.5% in adults^1^. The risk of malignancy is higher in deep masses^2,3^. A deep soft-tissue lesion is defined as one deep to the investing fascia or, in the hands and feet, deep to the palmar/plantar aponeuroses or subcutaneous tissues. Therefore, a deep anatomical location does not necessarily imply a deep physical location. For example, in the proximal arm or leg, a deep soft-tissue mass may be located either deep alongside the bone or superficially, within or just below the investing fascia^4–6^. Conversely in the hands and feet, anatomically deep soft tissue masses will be quite close to the skin^4–6^.

The initial diagnostic workup is crucial in diagnosing malignant soft-tissue tumors such as sarcomas^7^. Clinical examination can be limited to characterizing deep soft-tissue masses, for example, complementary imaging investigations are important in providing evidence for a potential malignant origin and guiding the need for a biopsy^8^. Radiological guidelines used to recommend magnetic resonance imaging (MRI) as the primary investigation method for deep soft-tissue masses. While MRI offers good contrast resolution for soft-tissue components, such as fat, muscle, no single MRI feature can reliably diagnose all soft-tissue tumors. Certain benign masses associated with inflammatory or traumatic etiologies may exhibit MRI signal intensity characteristics similar to those of malignant tumors^9^. However, significant advancements in ultrasound imaging techniques have been achieved in recent years. High-resolution probes, with a frequency of 10–15 MHz, enabled the acquisition of high-quality images, while low-frequency sector transducers can complement the examination of masses located deeper in the body, allowing a detailed study of the pediatric musculoskeletal system. Ultrasonography (US) has emerged as an appropriate and reliable diagnostic tool for several pediatric musculoskeletal pathologies. It is currently recommended as the initial imaging modality for children with soft tissue masses^10,11^. Deep and superficial masses can be distinguished using US because the spectrum of deep masses differs from that of superficial masses. A recent prospective study assessing the accuracy of US in characterizing 134 histologically confirmed deep soft-tissue masses demonstrated promising results. However, the average age of the patients in their study was over 50 years^5^.

Deep soft-tissue tumors in children are often documented in case reports or reports focused on individual diseases. Consequently, literature regarding the role of US as a primary diagnostic tool for deep soft-tissue tumors in children is limited. Hence, this study aimed to investigate the effectiveness of ultrasound in diagnosing deep soft-tissue tumors in children, including masses occurring on the trunk and extremities.

## Materials and methods

This study was approved by the Ethical Committee of West China Second University Hospital, Ethics No: (2023)35. Given that this study involved a noninvasive anonymous retrospective analysis, the requirement for written informed consent was waived by the Ethics Committee of the West China Second University Hospital of Sichuan University. The study was performed in accordance with all relevant guidelines and regulations.

### Patients

We conducted a retrospective review of electronic medical records in authors’ institution between January 2015 and December 2022 from pediatrics who were found deep soft masses with histological results. Demographic information data, including age and sex, presence of symptoms, history of malignancy, multiple occurrences and disease course of the patients were extracted for analysis. The disease course was defined as the interval from the initial detection of the mass to the first ultrasound examination. Histological findings of tumor lesions and vascular malformations were included in the analysis. Lymph nodes pathologies or hernias were not included^5,12,13^. Refer to the fifth edition of the World Health Organization Classification of Tumors of Soft Tissue and Bone published in early 2020^14^, pathological results were classified as benign group if ICD code was 0 or 1, or classified as malignant group if ICD code was 3, 6 or 9. Before surgery, MRI examinations were performed in 26 patients (28 lesions), after undergoing an ultrasound. The MRI results were not included in the statistical analysis because the information revealed on MRI was not blinded to the ultrasound findings and other patient data.

### Ultrasound examination and measurements

This study incorporated a GE Voluson E8 color Doppler multifunctional ultrasonic diagnostic instrument and Aplio 500 ultrasound scanner equipped with linear array probes. To ensure optimal image quality, parameters such as focus positions, brightness, gain, zoom, and depth were individually adjusted. Cine loops were recorded in the transverse and sagittal orientations within short video sequences. Radiologists captured all soft tissue tumors and their surrounding tissues. Cine loop acquisition was performed slowly, at a rate of approximately 15 s per loop, to avoid blurred images. No specific preparation was required prior to the examination. If children were crying and unable to undergo the examination, the ultrasound was performed when they were calm down. The ultrasound examinations were conducted before intervention by physicians with more than 3 years of experience. Color Doppler imaging was routinely performed, and the intensity of vascularity were assessed. Spectral analysis and sonoelastography were not routinely performed. Multi-section scanning technique was employed to observe and document various characteristics of the lesions, including number, size, echogenicity, shape, margin, calcification, and blood flow Adler grading. Multiple occurrences were defined as more than one soft tissue mass was observed. The echogenicity of the lesions was determined relative to adjacent muscle tissue and categorized as hypoechogenic, hyperechogenic, or isoechogenic. Three radiologists (Radiologists 1, 2, and 3) who were blinded to the medical history and other imaging findings of the patients independently reviewed the US studies, including images or saved cine loops. Radiologists 1, 2, and 3 had 2, 5, and 8 years of experience in pediatric ultrasound diagnosis, respectively. They diagnosed either benign or potentially malignant lesions based on their review of the ultrasound images.

### Statistical analysis

All statistical analyses were conducted using SPSS version 22.0 software (IBM Corporation, Armonk, NY, USA). The normality of data distribution was assessed using the Kolmogorov–Smirnov test. Non-normally distributed variables were compared using the Mann–Whitney *U* test and presented as medians with interquartile ranges. Categorical data were expressed as ratios and percentages. The χ^2^ test or Kruskal–Wallis *H* test was used to assess differences in proportions. Statistical significance was defined as p < 0.05. In cases where p < 0.10, a binary logistic regression analysis was performed to identify independent risk factors that could predict benign or malignant pathology.

### Ethics

Given that this study involved a noninvasive anonymous retrospective analysis, the requirement for written informed consent was waived by the Ethics Committee of the West China Second University Hospital of Sichuan University. The study was performed in accordance with all relevant guidelines and regulations.

## Results

A total of 74 children with 82 lesions were included in this study. Among the 74 children analyzed, 40 (54.1%) were male and 34 (45.9%) were female, with a median age of 36 (13, 73) months. No differences were observed between the benign and malignant subgroups in terms of age (p = 0.059) and sex (p = 1.0) (Table 1).

**Table 1:** Summary of study population characteristics.

	Malignant (n = 25)	Benign (n = 57)	p value
Sex (%)			
Female	11 (44.0%)	26 (45.6%)	1.0
Male	14 (56.0%)	31 (54.4%)	
Age, median (IQR) months	45 (24, 105)	32.0 (12.0, 66.0)	0.059
Presence of symptoms, n (%)			
Yes	3 (12.0%)	11 (19.3%)	0.534
No	22 (88.0%)	46 (80.7%)	
History of malignancy, n (%)			
Yes	8 (32.0%)	1 (1.8%)	0.000*
No	17 (68.0%)	56 (98.2%)	
Multiple occurrences, n (%)			
Yes	11 (44.0%)	3 (5.3%)	0.000*
No	14 (56.0%)	54 (94.7%)	
Disease course, n (%)			
< 3 months	15 (60.0%)	32 (56.1%)	0.812
≥ 3 months	10 (40.0%)	25 (43.9%)	

*Indicates that the p-value is less than 0.05.

Among the lesions, 47 (57.3%) were identified less than 3 months before surgery, while 35 (42.7%) were identified more than 3 months prior. No significant differences were observed in the disease course between the benign and malignant subgroups (p = 0.812). 68 (82.9%) lesions were solitary in the study. Four children had two deep soft-tissue lesions, and two children had three deep soft-tissue lesions. For instance, one patient was found to have two muscle tumors in the leg and back and a soft-tissue mass in the neck after birth, and the three masses were all diagnosed as indolent spindle cell tumor (an intermediate tumor, ICD-O-1). Upon histological examination, another patient had three deep masses in the buttocks, all of which were confirmed to be rhabdomyosarcoma. A statistically significant difference was observed between the isolated or multiple benign and malignant subgroups (p < 0.001). Table 2 presents the distribution of the lesion sites across the 82 cases, including various areas of the body involved in the study.

**Table 2:** Location of 82 deep soft-tissue lesions.

	Upper extremity (n = 12)	Lower extremity (n = 18)	Hand and wrist (n = 2)	Foot and ankle (n = 2)	Head and neck (n = 14)	Shoulder and axilla (n = 5)	Hips, groins, and buttocks (n = 5)	Trunk (n = 24)
Benign	10 (83.3%)	13 (72.2%)	2 (100.0%)	2 (100.0%)	6 (42.9%)	4 (80.0%)	2 (40.0%)	18 (75.0%)
Malignant	2 (16.7%)	5 (27.8%)	0 (0.0%)	0 (0.0%)	8 (57.1%)	1 (20.0%)	3 (60.0%)	6 (25.0%)

Fourteen patients presented with symptoms, primarily including pain or claudication. One male patient diagnosed with Langerhans cell hyperplasia exhibited fever. Among the two male patients diagnosed with Kaposi’s hemangioendothelioma, one experienced Carme’s phenomenon, which is characterized by fever and bleeding gums, while the other was without any symptom. No significant difference was observed between the benign and malignant subgroups regarding the presence or absence of symptoms (p = 0.534). Nine malignant lesions in this study were found to have a history of malignancy, all turned out to be the recurrence or metastasis of these primary tumors, including immature teratoma, lymphoma, and sarcoma. A statistically significant difference was observed in the history of malignancy between the benign and malignant subgroups (p < 0.001).

Out of 82 lesions analyzed, 25 (30.4%) were confirmed to be malignant based on histological examination, while 57 lesions (69.6%) were considered benign. The distribution of pathological types among the 82 lesions is provided in Table 3. Among the malignant cases, sarcoma was the most common (13/82, 15.9%), followed by metastatic tumors and lymphoma. In contrast, among the benign deep soft-tissue lesions, hemangiomas or vascular malformations were the most common (34/82, 41.4%), followed by adipocytic tumors. Seven cases of intermediate tumors with potential local invasiveness were identified among the benign lesions, including one cases of lipofibromatosis, two cases of Kaposiform haemangioendothelioma, one case of Langerhans cell histiocytosis and three cases of indolent spindle cell tumor. Among the six metastatic tumors identified, one originated from an immature teratoma of the ovary and was located in the rectus abdominis muscle.

**Table 3:** Pathology subtypes of 82 deep soft-tissue lesions.

	Pathology subtypes	Cases (%)
Benign	Vascular malformation/haemangioma	34 (41.4%)
	Adipocytic tumors	11 (13.4%)
	Tenosynovial giant cell tumor	1 (1.2%)
	Peripheral nerve sheath tumors	3 (3.7%)
	Perivascular tumors	1 (1.2%)
	Indolent spindle cell tumor*	3 (3.7%)
	Lipofibromatosis*	1 (1.2%)
	Kaposiform haemangioendothelioma*	2 (2.4%)
	Langerhans cell histiocytosis*	1 (1.2%)
Malignant	Sarcoma	13 (15.9%)
	Metastasis	6 (7.3%)
	Lymphoma	4 (4.9%)
	Rhabdoid tumor	2 (2.4%)

*Indicates that the ICD code was 1 (Intermediate tumors).

Table 4 presents the ultrasound parameters of the benign and malignant lesions. No statistically significant difference was observed in median maximum diameters (p = 0.359), margins (p = 1.0), calcification (p = 0.057), or blood Adler type (p = 0.563) between the benign and malignant groups. Significant differences were observed in terms of the shape and echogenicity type of the lesions between the benign and malignant groups (p < 0.001). Logistic regression analysis was performed, and tumor shape (p = 0.042, OR = 6.222), single or multiple occurrences (p = 0.008, OR = 17.000), and history of malignancy (p = 0.038, OR = 13.962) were identified as independent predictors of benign and malignant tumors (Table 5).

**Table 4:** Ultrasound parameters of benign and malignant tumors and tumor-like lesions.

	Malignant (n = 25)	Benign (n = 57)	p value
Maximum diameter, median (IQR) mm	40 (25.5, 46.5)	33.0 (23.5, 42.0)	0.359
Irregular shape, n (%)			
Yes	22 (88.0%)	26 (45.6%)	0.000*
No	3 (12.0%)	31 (54.4%)	
Ill-defined margin, n (%)			
Yes	11 (44.0%)	24 (42.1%)	1.0
No	14 (56.0%)	33 (57.9%)	
Calcification, n (%)			
Yes	7 (28.0%)	6 (10.5%)	0.057
No	18 (72.0%)	51 (89.5%)	
Echogenicity, n (%)			
Hypoecho	22 (88.0%)	12 (21.1%)	0.000*
Isoecho	0 (0.0%)	28 (49.1%)	
Hyperecho	3 (12.0%)	17 (29.8%)	
Blood Adler type, n (%)			
Grade 0	5 (20.0%)	14 (24.6%)	0.563
Grade I	8 (32.0%)	23 (40.3%)	
Grade II	9 (36.0%)	12 (21.1%)	
Grade III	3 (12.0%)	8 (14.0%)	

*Indicates that the p-value is less than 0.05.

**Table 5:** Result of logistic regression analysis.

Variable	Wald	OR	p value
Shape	4.128	6.222	0.042*
Echogenicity	2.091	0.441	0.148
Calcification	1.235	2.676	0.267
History of malignancy	4.27	13.962	0.038*
Age	0.849	0.357	1.007
Multiple	6.984	17.000	0.008*

*Indicates that the p-value is less than 0.05.

The statistical analysis included a comparison between diagnoses by radiologists and pathological results. The sensitivity, specificity, and kappa values are listed in Table 6. Radiologist 1 and Radiologist 2 misdiagnosed multiple rhabdomyosarcomas of the hip and a case of infantile fibrosarcoma in the chest wall as benign. In these cases, the maximum diameters of the three masses in the hip were 22, 9, and 6 mm, and 26 mm in the case of the chest wall. In the case of immature teratoma metastasis in the rectus abdominis muscle, three radiologists all misdiagnosed it as endometriosis. 4, 3, and 3 cases of the 34 hemangiomas/venous malformation lesions were misdiagnosed as malignant by radiologists 1, 2, and 3, and the diagnostic accuracy of three radiologists was 88.2%, 90.1%, and 90.1%, respectively. When diagnosing 11 adipocytic tumors, all three radiologists classified a case of plantar lipoblastoma as malignancy and correctly classified the rest 10 cases (diagnostic accuracy: 90.9%). In the seven cases of intermediate tumors, only one case(14.2%) of asymptomatic Kaposi haemangioendothelioma was correctly classified as benign by three radiologists, the other six cases(85.7%) were all classified as malignant.

**Table 6:** Diagnostic performance of three radiologists for identifying malignant from benign.

Reader	Sensitivity (%)	Specificity (%)	Kappa value
Radiologist 1	68.0	77.2	0.428
Radiologist 2	72.0	82.5	0.527
Radiologist 3	96.0	77.2	0.645

## Discussion

**Figure 1:**
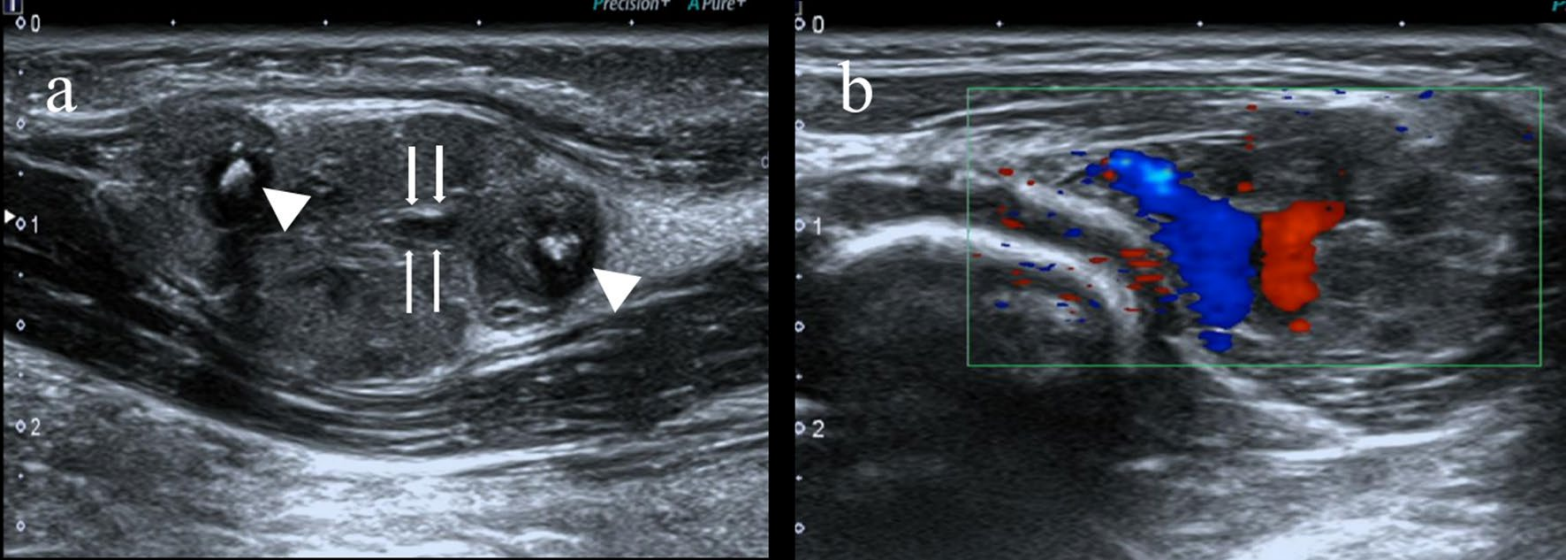
Asymptomatic intramuscular vein malformation was identified in a 3-year-old boy. (**a**) Transverse gray-scale US shows a slightly hyperechoic, reasonably well-defined mass located in the left upper arm biceps brachii with internal calcifications suspicious for phleboliths (arrowheads) and linear anechoic spaces (arrows) consistent with slow-flow venous channels. (**b**) Color Doppler flow imaging shows moderately central hyperemia.

Malignant soft-tissue masses are rare, with an average annual incidence of less than 1 per 1,000,000 children, excluding alveolar and embryonal rhabdomyosarcomas^15^. Among the 82 cases of masses included in this study, 25 cases of malignant lesions and seven cases of intermediate tumors with potential local invasiveness were identified among the benign lesions. Sarcomas accounted for the majority (52%) of the deep malignant masses observed, followed by metastatic tumors and lymphomas. Early detection and diagnosis are crucial in managing these conditions^16,17^. The earliest age at which the mass was discovered in this study was 17 days. Most identified masses were asymptomatic (Fig. 1), and only a small proportion of pediatric patients experienced pain or claudication. Patients often present to the clinic due to detecting lumps by their family members, particularly in cases where the language skill of the child is not fully developed.

Identifying deep soft-tissue tumors in adults using color Doppler ultrasonography typically relies on mass size, shape, and blood supply richness^4,17–20^. Morii et al. classified vascularity patterns into four types^20^, including type I (avascular), type II (hypovascular with a single vascular pole in the hilum), type III (hypervascular with multiple peripheral poles), or type IV (hypervascular with internal vessels). The longest diameter, tumor margin, and vascularity proved to be significantly different between benign and malignant tumor groups in their study. However, deep soft-tissue lesions in children exhibit unique ultrasonic characteristics. Our results showed no statistical differences in size and blood supply between benign and malignant tumors because young children have limited language development, and many of the detected lumps are asymptomatic, potentially leading to larger tumor sizes. Additionally, children have a higher incidence of vascular anomalies and benign vascular tumors, which can be large. Furthermore, the blood supply patterns of deep soft-tissue masses in children vary widely. Some vascular malformations and hemangiomas exhibit abundant blood supply, while others may demonstrate the opposite^18,20,21^. Malignant tumors may also exhibit diverse blood supply characteristics. Consequently, tumor size and blood supply richness may not be decisive factors distinguishing between benign and malignant deep lesions in pediatric patients.

**Figure 2:**
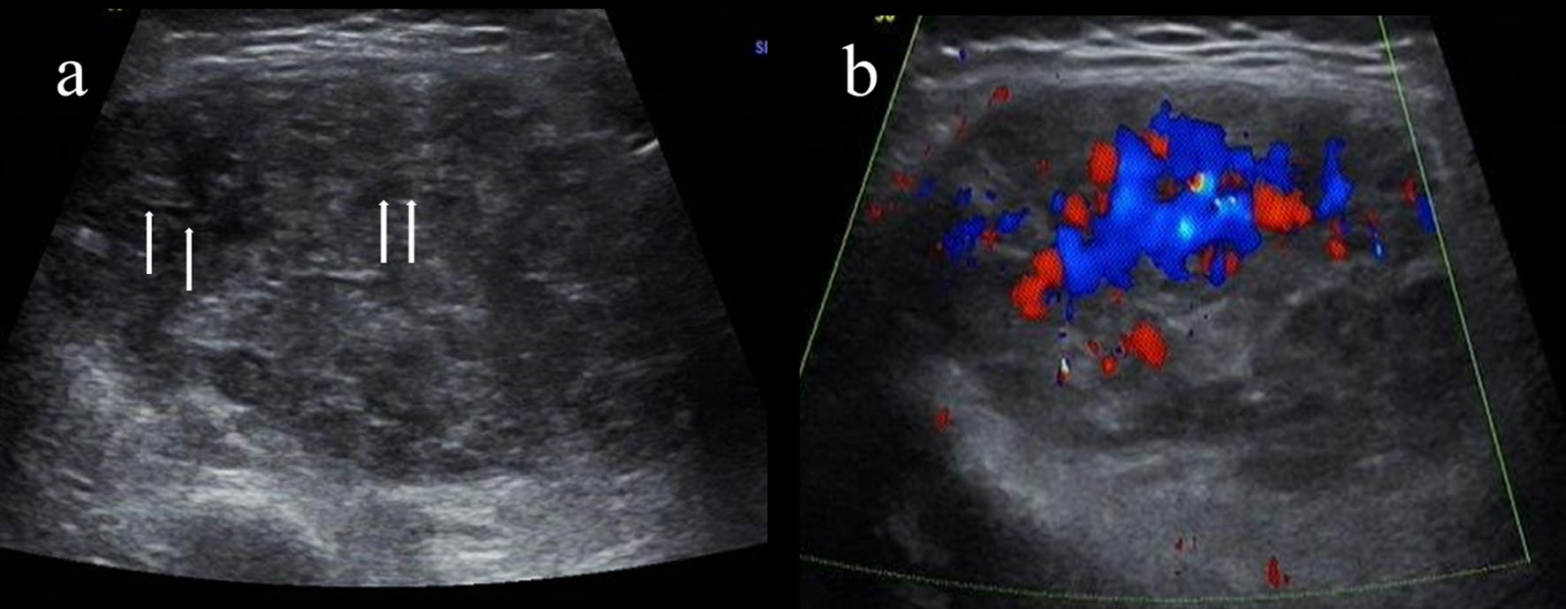
In a 2-year-old girl with claudication for a week, a mass was found in her right knee, which was later diagnosed as hemangioma. (**a**) Gray-scale US shows an ill-defined mass in the right knee with a maximum diameter of 52 mm. Linear anechoic spaces (arrows) are consistent with slow-flow venous channels. (**b**) Color doppler flow imaging shows rich central hyperemia.

**Figure 3:**
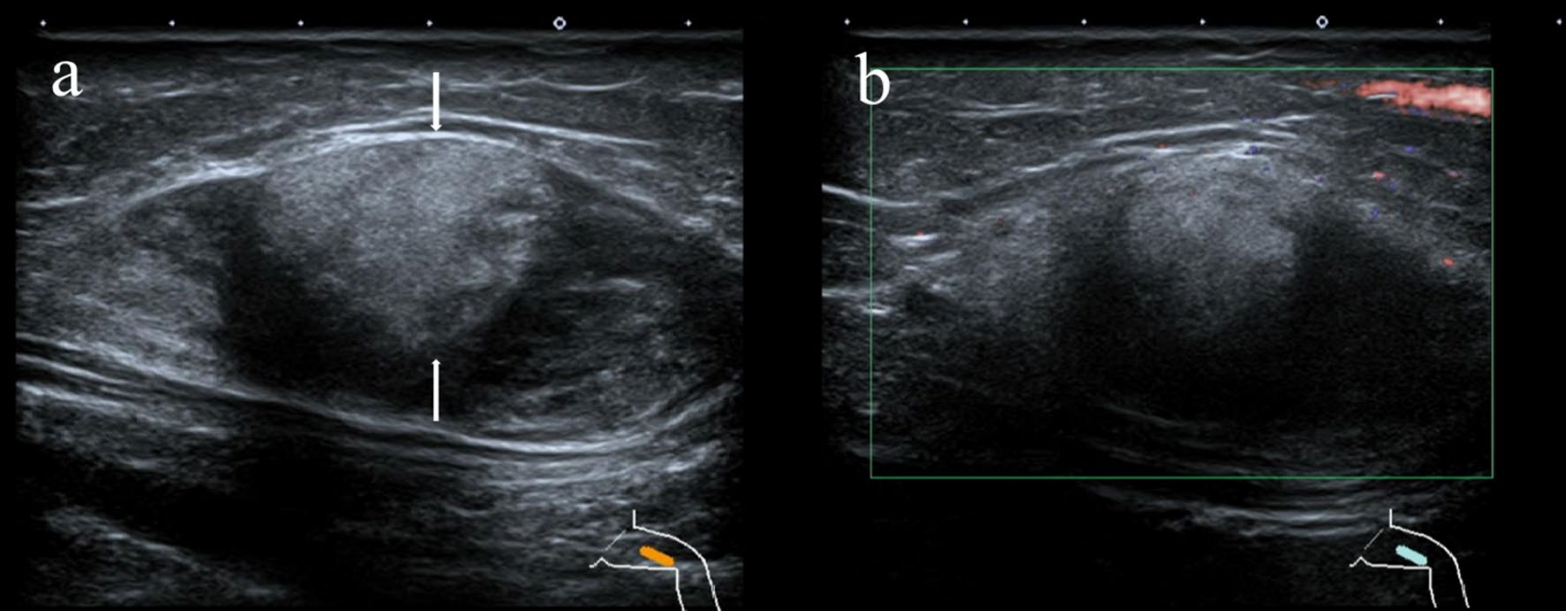
In a 4-year-old girl, an adipoblastoma was detected in her leg. (**a**) Gray-scale ultrasound examination showed a mixed echogenic, ill-defined mass lateral to the left sartorius muscle, with a patchy hyperechoic area (long arrow). The mass did not break through the deep fascia or invade the subcutaneous fat layer. (**b**) Color doppler flow imaging did not show an obvious blood flow signal within the mass.

**Figure 4:**
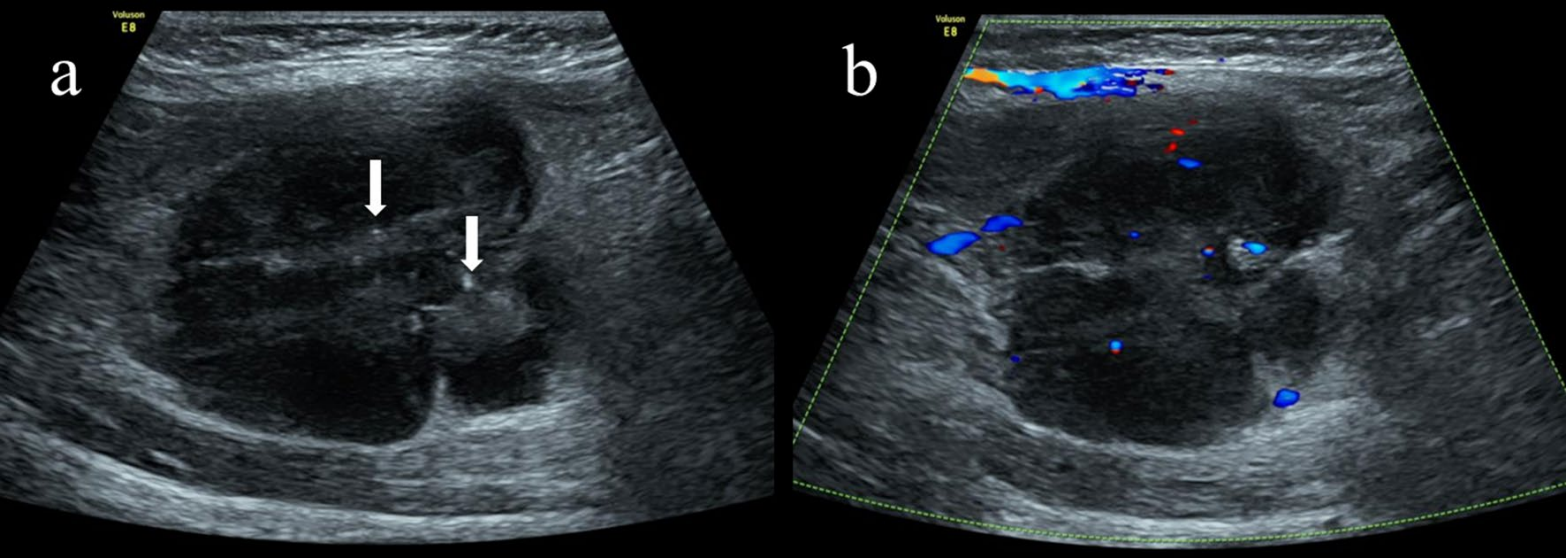
In a 1-year-old girl with a lump in her right leg, a histological examination confirmed the presence of embryonal rhabdomyosarcoma. (**a**) Transverse gray-scale US image of the right leg at the site with the boy in a supine position. Gray-scale ultrasound revealed a lobulated and well-defined mass with internal calcifications (arrows) located in the adductor muscle space of the thigh. The echo was lower than that of the adjacent muscle tissue. (**b**) Color doppler flow imaging indicated several punctured blood flow signals within the mass.

**Figure 5:**
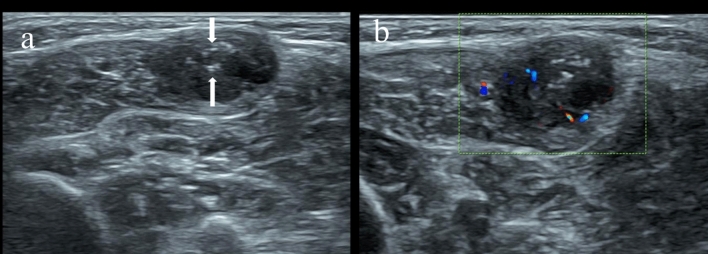
Depicts ultrasound images of a lump in the right calf of the same girl, as shown in Fig. 4, which was confirmed to be embryonal rhabdomyosarcoma through histological examination. (**a**) Gray-scale US image reveals a lobulated, well-defined hypoechoic mass with internal calcifications (arrows) within the gastrocnemius muscle. (**b**) Color doppler flow imaging indicates several punctured blood flow signals inside the mass.

Our findings indicate that tumor boundaries are unreliable indicators of benignity or malignancy. Certain benign tumors, including skeletal muscle vascular malformations (Fig. 2), neurofibromas, and adipoblastomas (Fig. 3), can exhibit poorly defined margins^18,21–23^, while sarcomas often display well-demarcated borders due to forming pseudo-capsules^24,25^ (Figs. 4 and 5). We did not observe a statistically significant difference in the presence of calcification between benign and malignant tumors. However, due to calcification presented in few cases, our classification did not distinguish between coarse or minimal calcification, and we also considered intralesional phleboliths as calcification. Previous studies have suggested that certain malignant masses, such as neuroblastomas, are more likely to exhibit calcification^25,26^. Therefore, radiologists should carefully evaluate calcification as it may carry diagnostic significance.

We observed that multiple deep soft-tissue tumors were associated with a higher likelihood of malignancy. Pathologists conducted comprehensive analyses of immunohistochemical results, gene mutation detection, and imaging findings for accurate diagnosis. Only one child was diagnosed with an indolent spindle cell tumor (an intermediate tumor, ICD-O-1) and did not experience recurrence during a 3-year follow-up period. Therefore, subjecting multiple deep soft-tissue masses to further histological examination is crucial to identify potential malignancies. Additionally, children with a history of malignancy have an increased risk of developing malignant tumors, such as rhabdomyosarcoma and anaplastic large-cell lymphoma^27,28^. Therefore, apart from ultrasound imaging, thorough investigation of the medical history of a patient by the radiologist is equally important in assessing the likelihood of malignancy.

Results from our study indicate that radiologists are more likely to misdiagnose small malignant deep soft tissue tumors as benign, especially by inexperienced radiologists. We also found that intermediate tumors were difficult to distinguish from malignant ones by ultrasound. Intermediate tumors are characterized by locally aggressive growth, which may present as destruction of surrounding tissues in ultrasound images^29^, and this has an impact on the diagnostic specificity. Besides, we found variability in diagnostic sensitivity among radiologists, which improved with accumulated work experience. Special training in pediatric radiology may be helpful to improve sensitivity and specificity. This applies both in terms of knowledge of differential diagnoses as well as performing sonography in children, such as technique issues. We also note that the proficiency and familiarity of the radiologists with specific tumors and their ultrasound features can influence diagnostic accuracy. Deep learning of ultrasound findings related to deep soft-tissue masses could potentially aid in diagnosis. Therefore, ultrasound findings of both benign and malignant tumors in deep soft tissues should be encouraged to be reported.

The pathological diagnosis of soft-tissue masses can pose challenges, particularly for atypical lesions which ultrasound features are not clearly benign. Radiologists play a crucial role in diagnosing typical diseases by combining clinical and imaging features. However, for atypical cases with potential malignancy, radiologists should strive to further clarify the characteristics of the images. Our study does not suggest that color Doppler ultrasound can replace MRI or enhanced CT for diagnosing deep soft-tissue masses in children, especially for cases where US fails to establish a specific diagnosis or accurately depict the margins of a soft-tissue mass. MRI has a unique advantage in staging large primary soft-tissue sarcomas and bone tumors with soft-tissue extension^30–32^. Enhanced CT effectively identifies atypical or large vascular malformations and assesses the extent of soft-tissue tumors, especially their relationship with adjacent bony structures and major vessels^33^. But three imaging modalities above have own advantages and disadvantages. In previous report, US can sometimes better determine the solid nature of lesions, which might appear misleadingly cystic or vascular on MRI if post-contrast imaging is not performed^34^. With the increasing use of contrast-enhanced ultrasound (CEUS) in adults and children, CEUS holds promise as an additional diagnostic tool for several pediatric musculoskeletal pathologies^35^. CEUS enables visualization of the microcirculation and can improve visualization in slow- or low-volume blood flow states, significantly improving the Doppler signal. The CEUS perfusion patterns were reported to relate to the underlying feature of tumor neoangiogenesis. They were of good predictive value for the characterization of STTs: having no contrast enhancement and having homogeneous contrast enhancement were related to benign STTs. In contrast, central defects and patchy enhancement were related to malignancy. CEUS, combined with conventional US, maybe a valuable technique for the differential diagnosis of soft-tissue tumors^21,36^.

Ultrasound diagnosis is based on comprehensive ultrasound image features and clinical data in clinical practice. The use of ultrasound techniques, such as compressing the masses with the probe to detect soft tissue masses (e.g., venous malformation), can enhance the sensitivity and specificity. Consequently, the sensitivity and specificity of the ultrasound diagnosis in this study may have been underestimated. In addition to using US as part of an initial workup of deep soft tissue lesions, radiologists should be aware of the complementary nature of US in the evaluation in pediatric deep soft lesions.

The current study had some limitations that should be acknowledged. First, as a retrospective study, inherent biases were observed due to the variability of ultrasound image acquisition by different physicians. Second, the lack of spectral Doppler data for many lesions limited the analysis of the value of spectral Doppler US in lesion identification. Spectral Doppler data can provide additional information about blood flow patterns and vascularity, which could have contributed to a more comprehensive analysis. Furthermore, the study had a relatively small sample size, which may limit the generalizability of the findings. Larger prospective studies must validate the results and provide more robust evidence.

## Conclusion

In conclusion, deep soft-tissue lesions in children have a higher likelihood of malignancy. Multiple irregular morphologies and a history of malignancy were identified as independent risk factors for malignant masses. Tumor size, boundary and blood supply may not the key to distinguish malignant and benign tumors in deep soft tissue. Ultrasound demonstrates good performance in the diagnosis of benign deep lesions such as hemangiomas/venous malformation and adipocytic tumors. The experience of radiologists in recognizing specific tumors is important. Careful attention should be paid to masses with ambiguous ultrasound features, as well as small lesions.

## Data Availability

The data that support the findings of this study are available from the corresponding author but restrictions apply to the availability of these data, which were used under license from the West China Second University Hospital (China) for the current study, and so are not publicly available. Data are, however, available from the corresponding author upon reasonable request and with permission from the West China Second University Hospital (China).
